# Exploring the Link between BMI and Aggressive Histopathological Subtypes in Differentiated Thyroid Carcinoma—Insights from a Multicentre Retrospective Study

**DOI:** 10.3390/cancers16071429

**Published:** 2024-04-07

**Authors:** Giacomo Di Filippo, Gian Luigi Canu, Giovanni Lazzari, Dorin Serbusca, Eleonora Morelli, Paolo Brazzarola, Leonardo Rossi, Benard Gjeloshi, Mariangela Caradonna, George Kotsovolis, Ioannis Pliakos, Efthymios Poulios, Theodosios Papavramidis, Federico Cappellacci, Pier Francesco Nocini, Pietro Giorgio Calò, Gabriele Materazzi, Fabio Medas

**Affiliations:** 1 Endocrine Surgery Unit, Department of Surgery and Oncology, Verona University Hospital, 37134 Verona, Italy; 2 Department of Surgical Sciences, University of Cagliari, SS554 Bivio Sestu, Monserrato, 09042 Cagliari, Italy; 3 Endocrine Surgery Unit, University Hospital of Pisa, Via Paradisa 2, 56100 Pisa, Italy; 4 First Propedeutic Department of Surgery, AHEPA University Hospital, Aristotle University of Thessaloniki, 85 Karakasi Str., 54453 Thessaloniki, Greece; 5 Department of Oral and Maxillofacial Surgery, University of Verona, 37134 Verona, Italy

**Keywords:** thyroid cancer, aggressive subtypes, papillary thyroid cancer, obesity, body mass index

## Abstract

**Simple Summary:**

Our study aimed to investigate the suggested association between body mass index and aggressive histopathological subtypes of thyroid cancer. Thus, we studied 3868 patients who underwent thyroidectomy from 2020 to 2022 at four European centres. We found that overweight and obese patients with papillary thyroid carcinoma had higher rates of aggressive histopathological subtypes, bilateral, multifocal tumours, and larger nodal metastases. These findings suggest that people with higher body mass index may be at an increased risk of developing more aggressive features of thyroid cancer.

**Abstract:**

Obesity’s role in thyroid cancer development is still debated, as well as its association with aggressive histopathological subtypes (AHSs). To clarify the link between Body Mass Index (BMI) and AHS of differentiated thyroid carcinoma (DTC), we evaluated patients who underwent thyroidectomy for DTC from 2020 to 2022 at four European referral centres for endocrine surgery. Based on BMI, patients were classified as normal-underweight, overweight, or obese. AHSs were defined according to 2022 WHO guidelines. Among 3868 patients included, 34.5% were overweight and 19.6% obese. Histological diagnoses were: 93.6% papillary (PTC), 4.8% follicular (FTC), and 1.6% Hürthle cell (HCC) thyroid carcinoma. Obese and overweight patients with PTC had a higher rate of AHSs (*p* = 0.03), bilateral, multifocal tumours (*p* = 0.014, 0.049), and larger nodal metastases (*p* = 0.017). In a multivariate analysis, BMI was an independent predictor of AHS of PTC, irrespective of gender (*p* = 0.028). In younger patients (<55 years old) with PTC > 1 cm, BMI predicted a higher ATA risk class (*p* = 0.036). Overweight and obese patients with FTC had larger tumours (*p* = 0.036). No difference was found in terms of AHS of FTC and HCC based on BMI category. Overweight and obese patients with PTC appear to be at an increased risk for AHS and aggressive clinico-pathological characteristics.

## 1. Introduction

Thyroid cancer (TC) is an increasingly prevalent disease, particularly in high-income countries, and is projected to become the fourth most common cancer in the United States by 2030 [1,2]. Environmental and socio-demographic factors, including higher body mass index (BMI) and obesity, have been hypothesised to be linked to this surge in TC incidence [3,4,5,6]. Indeed, obesity, a global epidemic affecting 59% of Europeans, has been causally associated with 13 types of cancers, contributing to approximately 200,000 new cases annually [7,8,9,10]. The biological plausibility of obesity’s role in thyroid carcinogenesis has been speculated to involve low-grade chronic inflammation, altered cytokine levels, and increased oxidative stress found in this condition. Insulin resistance and hormonal changes, part of the pathological landscape of obesity, may also play a pivotal role [11,12,13]. However, obesity’s impact on aggressive clinico-pathological characteristics of differentiated thyroid cancer (DTC) remains unclear. Indeed, while some studies have suggested an association between higher BMI and aggressive tumour features of DTC, others have failed to demonstrate such a correlation [14,15,16,17,18]. Conversely, studies exploring the possible link between BMI and aggressive histopathological subtypes of DTC are currently lacking. Identifying TCs with aggressive histology or clinico-pathological characteristics that increase the risk of progression or relapse is crucial for directing therapeutic efforts more effectively and ensuring proper resource management.

This study aimed to assess BMI as a potential risk factor for aggressive DTC subtypes or clinico-pathological characteristics.

## 2. Materials and Methods

### 2.1. Study Design and Patient Selection

We conducted a multicentre retrospective cohort study including patients with a histopathologically confirmed diagnosis of DTC who underwent surgery between January 2020 and December 2022 at 4 european tertiary referral centres for endocrine surgery: Endocrine Surgery Unit—Verona University Hospital (Verona, Italy), Endocrine Surgery Unit—Pisa University Hospital (Pisa, Italy), General Surgery Unit—Cagliari University Hospital (Cagliari, Italy), and 1st Propaedeutic Department of Surgery—AHEPA University Hospital (Thessaloniki, Greece). 

The patients included in the present study underwent either hemithyroidectomy, total thyroidectomy, or completion thyroidectomy with or without lymphadenectomy. Patients younger than 18, with incomplete data or with a histopathological diagnosis of anaplastic or poorly differentiated TC, medullary TC, thyroid lymphoma or metastasis were excluded from the study. Patients who underwent lobectomy and subsequent completion thyroidectomy were considered as a single case for the purposes of this analysis.

A written informed consent to anonymised data collection was signed by each patient included in the study. 

The present study is in accordance with the ethical standards of the 1964 Helsinki Declaration and its later amendments or comparable ethical standards.

### 2.2. Data Collection

Patients’ clinical data were collected from computerised medical charts and entered into an anonymized database. Data collected included: patient’s age at surgery, gender, BMI, preoperative diagnosis, presence of hyperthyroidism or thyroiditis, type of surgery, number of excised and pathological lymph nodes, histopathological diagnosis, neoplasm diameter, histological variant, multifocality and bilaterality, surgical margin status, vascular infiltration, extrathyroid extension, and American Thyroid Association (ATA) risk score for disease recurrence [19]. 

Based on BMI, patients were classified as normal-underweight (<25 kg/m^2^), overweight (25–29.9 kg/m^2^), or obese (>29.9 kg/m^2^) according to WHO guidelines [20]. 

Histopathological subtypes and features of DTC were classified as aggressive (aggressive histopathological subtype, AHS) based on the latest WHO guidelines for TC classification [21], i.e., according to the following criteria: tall cell PTC (proportion of subtype features ≥30% of total); hobnail PTC (proportion of subtype features ≥30% of total); solid PTC (proportion of subtype features ≥50% of total); columnar cell PTC; diffuse sclerosing PTC; extensively invasive FTC; or angioinvasive FTC with >4 invasion foci.

### 2.3. Statistical Analysis

Continuous variables were expressed as median values and interquartile ranges [IQR], while categorical variables were presented as frequencies and percentages.

Collected sociodemographic and histopathological characteristics were compared between BMI categories using Mann–Whitney, Kruskal–Wallis, and Chi Square tests as appropriate.

Differences in histopathological features between different BMI categories were tested separately for patients with papillary thyroid cancer (PTC), follicular thyroid cancer (FTC), and oncocytic thyroid cancer (HCC).

A multivariate binary logistic regression analysis was performed to test whether BMI represented an independent predictor of AHS using preoperative data as confounders. 

For all tests, a *p*-value < 0.05 was considered statistically significant. Statistical analysis was performed using SPSS (Statistical Package for the Social Sciences, IBM SPSS Statistics for Windows, Version 25.0. IBM Corp.: Armonk, NY, USA).

## 3. Results

Out of 3925 patients meeting the inclusion criteria, 57 were excluded from the analysis due to missing data. Consequently, the final analysis included 3868 patients.

**Figure 1:**
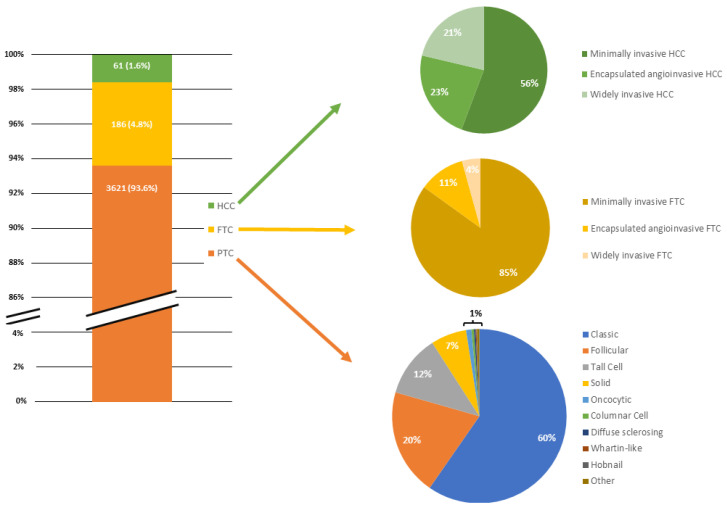
Bar chart and pie charts depicting the relative proportion of differentiated thyroid cancers included in the study and each histopathologic subtype within each neoplasm.

Sociodemographic and clinicopathological characteristics of the study population are summarised in Table 1 and Table 2 and Figure 1.

Among the 3868 patients included, 2765 (71.5%) were female. The median BMI was 25 kg/m^2^ (IQR 22–28) with 1333 patients (34.5%) classified as overweight and 757 (19.6%) as obese. Histological diagnoses revealed 93.6% PTC, 4.8% FTC, and 1.6% HCC. Nearly 47% of patients underwent surgery with a preoperative diagnosis of malignancy. Total thyroidectomy was performed in 84.5% of cases while 12.7% underwent lobectomy. Central compartment lymphadenectomy and lateral compartment dissection were performed in 20% and 8.2% of patients, respectively.

Differences between histopathological features among BMI categories are summarised in Table 3, Table 4 and Table 5 for PTC, FTC, and HCC, respectively.

Obese and overweight patients with PTC were older (52 and 53 vs. 46 years old; *p* < 0.0005) and more frequently male (37.3% and 31.6% vs. 20.1%; *p* < 0.0005) than normal/underweight patients. Obese patients had a higher rate of AHS (22.3% vs. 18.6%; *p* = 0.03), bilateral (30.9% vs. 25.6%; *p* = 0.014), multifocal tumours (32.4% vs. 28.2%, *p* = 0.049), and larger nodal metastases (8 mm vs. 6 mm; *p* = 0.017) than normal/underweight patients. In the multivariate analysis, BMI was found to be an independent predictor of AHS of PTC, irrespective of gender (B = 0.018, *p* = 0.028) (Table 6). In younger patients (<55 years old) with PTC > 1 cm, a higher BMI predicted a higher ATA risk class (B = 0.02, *p* = 0.036). Overweight and obese patients with FTC had larger tumours (*p* = 0.036). No difference was found in terms of aggressive histopathological features of FTC and HCC based on BMI categories.

## 4. Discussion

Recent evidence has suggested that obesity may increase the risk of various cancers, including TC. However, the specific role of individual obesity-related factors in carcinogenesis remains uncertain [12,22,23]. The association between BMI and TC is believed to be linked to shared hormonal and metabolic factors related to central adiposity, as well as potential interactions with genetic variants of the fat mass and obesity-associated (FTO) gene. Certain FTO gene variants, particularly in combination with higher BMI, have been associated with an elevated risk of TC [24]. Moreover, obesity itself may contribute to chronic low-grade inflammation and altered insulin signalling, promoting tumorigenesis [8]. However, the current understanding lacks data on the correlation between BMI and aggressive histopathological subtypes of thyroid cancer.

Our study identified significant associations between BMI and the AHS of PTC. Overweight and obese patients exhibited a higher proportion of AHSs of PTC compared to their normal/underweight counterparts. This association was consistent across genders.

In other cancer types, BMI has been identified as a risk factor for the emergence of more aggressive subtypes. For instance, in premenopausal women, obesity is associated with an elevated risk of the triple-negative breast cancer subtype and non-luminal subtypes [25,26]. Similarly, a high BMI is linked to an increased risk of borderline serous, invasive endometrioid, and invasive mucinous ovarian cancer subtypes [27]. The authors postulated a potential correlation between different cancer subtypes and the inflammatory adipose microenvironment rich in IL-6 and TNF-alpha, along with heightened levels of IGF-1 observed in obese patients. We speculate that similar molecular pathways may play a role in the development of distinct and more aggressive subtypes of PTC in obese individuals. Such molecular pathways may either act independently or interact with other known drivers of PTC tumorigenesis exacerbating tumor aggressiveness in obese individuals. Further in vivo and in vitro studies are needed to investigate the potential effects of adipose-tissue-derived factors on PTC tumorigenesis in obese patients.

Our data indicate that BMI could serve as a predictor of AHS, irrespective of gender. While the strength of the association is modest, we believe that clinicians should not overlook this finding and should consider incorporating BMI monitoring as part of the routine risk assessment for PTC.

In our study, overweight/obese patients with PTC had a higher proportion of bilateral, multifocal tumours, and larger nodal metastases than normal/underweight patients. Additionally, in younger patients (<55 years old) with PTC > 1 cm, the BMI predicted a higher ATA risk class. These associations were not observed in patients with FTC and HCC.

Studies investigating the relationship between BMI and aggressive histopathological features of TC have yielded conflicting results. While some studies have found no positive association between BMI and aggressive tumour features or recurrence [14,28], others have reported a significant association between higher BMI and extrathyroidal extension, multifocality, and lymph node metastasis in PTC [15,16,29]. Recent evidence suggests that obese patients with TC may activate different pathways compared to normal-weight patients. In a study by Basolo et al. [30], genes involved in metabolic pathways and immune-cell-related mechanisms were expressed differently in the thyroid tissue of obese patients compared to normal-weight patients. Furthermore, in a study on murine animal models by Kim et al., obesity exacerbated TC progression, resulting in increased tumour growth and a more aggressive type of TC [31].

We hypothesise that obesity may be a potential risk factor for the development of aggressive clinicopathological features in PTC, especially in younger patients. Although the exact mechanisms are not fully understood, it is conceivable that specific molecular pathways and gene expression profiles within adipose tissue, along with low-grade chronic inflammation, could play a role in the emergence of these aggressive features in PTC.

The lack of similar associations in patients with FTC and HCC may be attributed to various factors. We can speculate that the molecular mechanisms leading to the expression of aggressive features in TC among obese individuals could be specific to PTC. Additionally, the relatively small sample size of FTC and HCC patients should be considered, potentially impacting the ability to identify comparable associations in these subgroups. Furthermore, the retrospective nature of our study introduces inherent selection bias, potentially limiting the generalizability of these findings to a broader population. Lastly, in the present study, BMI was used as the primary metric for assessing obesity and overweight status, according to WHO definitions. However, although BMI is a widely accepted and practical measure, future research exploring obesity-related associations with cancer subtypes may also benefit from considering additional measures to provide a more comprehensive evaluation.

A significant strength of this study lies in the inclusion of a multicentric, large, diverse and representative sample, enhancing the external validity of our findings. Furthermore, the robustness of our study is underscored by a meticulous data collection process that systematically included a wide range of histopathological features in the analysis. This comprehensive approach contributed to a more nuanced understanding of the subject and improved our possibilities of identifying meaningful associations within the data.

Although our study supports the correlation between BMI and aggressive histopathological variants, further multicentric prospective studies with homogeneous samples are needed to confirm our results.

## 5. Conclusions

Our study contributes insights into the relationship between obesity and DTC, specifically focusing on the potential role of BMI in predicting AHS and aggressive clinico-pathological features of PTC. Caution should be used in generalizing these results to other TC subtypes, as the molecular dynamics may vary. Prospective studies are needed to confirm our findings.

## Figures and Tables

**Table 1:** Sociodemographic and surgical characteristics of the whole population.

Variable		N (%); Median (IQR)
Age at Surgery, years		50 (38–60)
BMI, kg/m^2^		25 (22–28)
BMI, kg/m^2^	<25	1778 (46%)
	25–29.9	1333 (34.5%)
	>29.9	757 (19.6%)
Gender	Female	2765 (71.5%)
	Male	1103 (28.5%)
Hyperthyroidism	No	3486 (90.1%)
	Yes	382 (9.9%)
Preoperative Diagnosis	Basedow	141 (3.6%)
	Indeterminate nodule	1026 (26.5%)
	Malignancy	1806 (46.7%)
	N/MNG	885 (22.9%)
	Plummer	10 (0.3%)
Substernal Goiter	No	3765 (97.3%)
	Yes	103 (2.7%)
Type of Surgery	Completion Thyroidectomy	29 (0.7%)
	Lobectomy	492 (12.7%)
	Lobectomy + Completion Thyroidectomy	77 (2%)
	Total Thyroidectomy	3270 (84.5%)
Monolateral Central Compartment lymphadenectomy	No	3847 (99.5%)
	Yes	21 (0.5%)
Bilateral Central Compartment lymphadenectomy	No	3115 (80.5%)
	Yes	753 (19.5%)
Monolateral Lateral Compartment lymphadenectomy	No	3586 (92.7%)
	Yes	282 (7.3%)
Bilateral Lateral Compartment lymphadenectomy	No	3835 (99.1%)
	Yes	33 (0.9%)

BMI, Body Mass Index; N/MNG, nodular/multinodular goiter; IQR, Interquartile Range.

**Table 2:** Pathological characteristics of the whole population.

Variable		N (%);
		Median (IQR)
Chronic Thyroiditis	No	2407 (62.2%)
	Yes	1461 (37.8%)
Histopathology	FTC	186 (4.8%)
	HCC	61 (1.6%)
	PTC	3621 (93.6%)
Max Cancer Diameter, mm		11 (5–19)
N° microfoci		2 (1–2)
Lymph Node Metastasis	No	655 (47.6%)
	Yes	720 (52.4%)
CC Pathological Lymph Nodes	No	689 (51.3%)
	Yes	654 (48.7%)
CC N lymph nodes excised		5 (2–9)
CC N Pathological Lymph Nodes		0 (0–3)
LC Pathological Lymph Nodes	No	308 (51.2%)
	Yes	294 (48.8%)
LC N lymph nodes excised		23 (16–31)
LC N Pathological Lymph Nodes		0 (0–3)
Pathological lymph node max dimension, mm		8 (3–16)
Extranodal infiltration	No	3094 (97.7%)
	Yes	72 (2.3%)
Aggressive Variant	No	3151 (81.5%)
	Yes	717 (18.5%)
Multifocal	No	2164 (55.9%)
	Yes	1704 (44.1%)
Bilateral	No	2734 (72.9%)
	Yes	1016 (27.1%)
Aggressive Variant on Microfoci	No	1648 (90.6%)
	Yes	171 (9.4%)
Surgical Margin Infiltration	No	3828 (99%)
	Yes	40 (1%)
Extrathyroid Microscopic infiltration	No	3095 (80%)
	Yes	773 (20%)
Extrathyroid Macroscopic Infiltration	No	3785 (97.9%)
	Yes	83 (2.1%)
Vascular-Lymphatic infiltration	No	3249 (84%)
	Yes	619 (16%)
Metastasis	No	3606 (99.9%)
	Yes	1 (0.1%)
pT	1A	1822 (47.1%)
	1B	1147 (29.7%)
	2	597 (15.4%)
	3A	222 (5.7%)
	3B	57 (1.5%)
	4A	21 (0.5%)
pN	0	655 (47.6%)
	1A	426 (31%)
	1B	294 (21.4%)
pM	0	371 (99.7%)
	1	1 (0.3%)
ATA Risk stratification system	High	395 (10.2%)
	Intermediate	1386 (35.8%)
	Low	2087 (54%)

IQR, Interquartile Range; FTC, Follicular Thyroid Carcinoma; HCC, Oncocytic Cell Carcinoma; PTC, Papillary Thyroid Carcinoma; CC, Central Compartment; LC, Lateral Compartment; ATA, American Thyroid Association.

**Table 3:** Differences in sociodemographic and pathological characteristics of PTC patients between BMI categories.

		BMI, kg/m^2^			
		<25	25–29.9	>29.9	
		N (%); Median (IQR)	N (%); Median (IQR)	N (%); Median (IQR)	*p* Value
Age at Surgery, years		46 (35–57)	52 (41–62)	53 (42–61)	0.001
Gender	Female	1330 (79.9%)	779 (62.7%)	488 (68.4%)	0.001
	Male	335 (20.1%)	464 (37.3%)	225 (31.6%)	
Hyperthyroidism	No	1505 (90.4%)	1109 (89.2%)	639 (89.6%)	0.570
	Yes	160 (9.6%)	134 (10.8%)	74 (10.4%)	
Preoperative Diagnosis	Basedow	67 (4%)	46 (3.7%)	24 (3.4%)	0.001
	Indeterminate nodule	394 (23.7%)	307 (24.7%)	167 (23.4%)	
	Malignancy	904 (54.3%)	569 (45.8%)	307 (43.1%)	
	Nodular or multinodular Goiter	295 (17.7%)	321 (25.8%)	210 (29.5%)	
	Plummer	5 (0.3%)	-	5 (0.7%)	
Substernal Goiter	No	1639 (98.4%)	1212 (97.5%)	684 (95.9%)	0.001
	Yes	26 (1.6%)	31 (2.5%)	29 (4.1%)	
Type of Surgery	Completion Thyroidectomy	8 (0.5%)	14 (1.1%)	3 (0.4%)	0.870
	Lobectomy	220 (13.2%)	137 (11%)	75 (10.5%)	
	Lobectomy + Completion Thyroidectomy	37 (2.2%)	17 (1.4%)	15 (2.1%)	
	Total Thyroidectomy	1400 (84.1%)	1075 (86.5%)	620 (87%)	
Monolateral Central Compartment lymphadenectomy	No	1655 (99.4%)	1237 (99.5%)	710 (99.6%)	0.820
	Yes	10 (0.6%)	6 (0.5%)	3 (0.4%)	
Bilateral Central Compartment lymphadenectomy	No	1297 (77.9%)	1007 (81%)	572 (80.2%)	0.100
	Yes	368 (22.1%)	236 (19%)	141 (19.8%)	
Monolateral Lateral Compartment lymphadenectomy	No	1535 (92.2%)	1143 (92%)	662 (92.8%)	0.770
	Yes	130 (7.8%)	100 (8%)	51 (7.2%)	
Bilateral Lateral Compartment lymphadenectomy	No	1653 (99.3%)	1230 (99%)	706 (99%)	0.610
	Yes	12 (0.7%)	13 (1%)	7 (1%)	
Chronic Thyroiditis	No	986 (59.2%)	777 (62.5%)	463 (64.9%)	0.021
	Yes	679 (40.8%)	466 (37.5%)	250 (35.1%)	
Variant	Classic	1026 (61.6%)	731 (58.8%)	403 (56.5%)	0.012
	Columnar Cell	3 (0.2%)	5 (0.4%)	9 (1.3%)	
	Diffuse sclerosing	5 (0.3%)	2 (0.2%)	4 (0.6%)	
	Follicular	301 (18.1%)	272 (21.9%)	143 (20.1%)	
	Hobnail	3 (0.2%)	-	-	
	Oncocytic	19 (1.1%)	12 (1%)	5 (0.7%)	
	Other	8 (0.5%)	7 (0.6%)	1 (0.1%)	
	Solid	115 (6.9%)	71 (5.7%)	53 (7.4%)	
	Tall Cell	183 (11%)	141 (11.3%)	93 (13%)	
	Whartin-like	2 (0.1%)	2 (0.2%)	2 (0.3%)	
Aggressive Variant	No	1356 (81.4%)	1024 (82.4%)	554 (77.7%)	0.033
	Yes	309 (18.6%)	219 (17.6%)	159 (22.3%)	
Aggressive Variant on Microfoci	No	703 (90.6%)	558 (93.2%)	328 (87.2%)	0.008
	Yes	73 (9.4%)	41 (6.8%)	48 (12.8%)	
Max Cancer Diameter		11 (6–18)	10 (4–17)	11 (5–17)	0.378
Multifocal Tumor	No	1195 (71.8%)	849 (68.3%)	482 (67.6%)	0.049
	Yes	470 (28.2%)	394 (31.7%)	231 (32.4%)	
N microfoci		1 (1–2)	2 (1–3)	2 (1–3)	0.011
AHS on main tumor OR on microfoci	No	1333 (80.1%)	1015 (81.7%)	539 (75.6%)	0.005
	Yes	332 (19.9%)	228 (18.3%)	174 (24.4%)	
Bilateral	No	1199 (74.4%)	856 (70.6%)	477 (69.1%)	0.014
	Yes	413 (25.6%)	357 (29.4%)	213 (30.9%)	
Lymph Node Metastasis	No	287 (44.6%)	185 (46.3%)	120 (46%)	0.840
	Yes	357 (55.4%)	215 (53.8%)	141 (54%)	
CC Pathological Lymph Nodes	No	300 (47.8%)	198 (50.5%)	127 (50.2%)	0.640
	Yes	328 (52.2%)	194 (49.5%)	126 (49.8%)	
CC N lymph nodes excised		5 (3–9)	6 (2–10)	5 (2–9)	0.313
CC N Pathological Lymph Nodes		1 (0–3)	0 (0–3)	0 (0–3)	0.778
LC Pathological Lymph Nodes	No	122 (47.8%)	71 (40.8%)	51 (47.7%)	0.310
	Yes	133 (52.2%)	103 (59.2%)	56 (52.3%)	
LC N lymph nodes excised		23 (17–31)	23 (16–32)	24 (17–35)	0.556
LC N Pathological Lymph Nodes		1 (0–4)	1 (0–4)	1 (0–3)	0.211
Pathological lymph node max dimension		6 (3–15)	10 (3.5–20)	8 (2–15.5)	0.017
Extranodal infiltration	No	1326 (97.5%)	988 (97.7%)	574 (97.5%)	0.920
	Yes	34 (2.5%)	23 (2.3%)	15 (2.5%)	
Surgical Margin Infiltration	No	1649 (99%)	1226 (98.6%)	706 (99%)	0.540
	Yes	16 (1%)	17 (1.4%)	7 (1%)	
Extrathyroid Microscopic infiltration	No	1317 (79.1%)	980 (78.8%)	560 (78.5%)	0.950
	Yes	348 (20.9%)	263 (21.2%)	153 (21.5%)	
Extrathyroid Macroscopic Infiltration	No	1632 (98%)	1216 (97.8%)	694 (97.3%)	0.570
	Yes	33 (2%)	27 (2.2%)	19 (2.7%)	
Vascular-Lymphatic infiltration	No	1404 (84.3%)	1066 (85.8%)	619 (86.8%)	0.240
	Yes	261 (15.7%)	177 (14.2%)	94 (13.2%)	
Metastasis	No	1563 (100%)	1164 (100%)	642 (99.8%)	0.120
	Yes	-	-	1 (0.2%)	
pT	1A	819 (49.2%)	625 (50.3%)	357 (50.1%)	0.160
	1B	512 (30.8%)	368 (29.6%)	207 (29%)	
	2	247 (14.8%)	167 (13.4%)	88 (12.3%)	
	3A	56 (3.4%)	57 (4.6%)	42 (5.9%)	
	3B	22 (1.3%)	19 (1.5%)	12 (1.7%)	
	4A	8 (0.5%)	6 (0.5%)	7 (1%)	
pT	pT1 or pT2	1578 (94.8%)	1160 (93.3%)	652 (91.4%)	0.008
	pT3 or pT4	87 (5.2%)	83 (6.7%)	61 (8.6%)	
pN	0	287 (44.6%)	185 (46.3%)	120 (46%)	0.150
	1A	224 (34.8%)	112 (28%)	85 (32.6%)	
	1B	133 (20.7%)	103 (25.8%)	56 (21.5%)	
M	0	154 (100%)	83 (100%)	54 (98.2%)	0.115
	1	-	-	1 (1.8%)	
ATA Risk stratification system	High	175 (10.5%)	122 (9.8%)	71 (10%)	0.042
	Intermediate	638 (38.3%)	413 (33.2%)	258 (36.2%)	
	Low	852 (51.2%)	708 (57%)	384 (53.9%)	

BMI, Body Mass Index; N/MNG, nodular/multinodular goiter; CC, Central Compartment; LC, Lateral Compartment; ATA, American Thyroid Association.

**Table 4:** Differences in sociodemographic and pathological characteristics of FTC patients between BMI categories.

		BMI, kg/m^2^			
		<25	25–29.9	>29.9	
		N (%); Median (IQR)	N (%); Median (IQR)	N (%); Median (IQR)	*p* Value
Age at Surgery, years		50 (36–63)	54 (46–67)	53.5 (44–60.5)	0.280
Gender	Female	70 (78.7%)	42 (60.9%)	16 (57.1%)	0.020
	Male	19 (21.3%)	27 (39.1%)	12 (42.9%)	
Hyperthyroidism	No	83 (93.3%)	62 (89.9%)	28 (100%)	0.205
	Yes	6 (6.7%)	7 (10.1%)	-	
Preoperative Diagnosis	Basedow	3 (3.4%)	1 (1.4%)	-	0.583
	Indeterminate nodule	61 (68.5%)	40 (58%)	16 (57.1%)	
	Malignancy	5 (5.6%)	6 (8.7%)	3 (10.7%)	
	N/MNG	20 (22.5%)	22 (31.9%)	9 (32.1%)	
	Plummer	-	-	-	
Substernal Goiter	No	86 (96.6%)	60 (87%)	25 (89.3%)	0.070
	Yes	3 (3.4%)	9 (13%)	3 (10.7%)	
Type of Surgery	Completion Thyroidectomy	2 (2.2%)	1 (1.4%)	-	0.639
	Lobectomy	28 (31.5%)	14 (20.3%)	7 (25%)	
	Lobectomy + Completion Thyroidectomy	3 (3.4%)	1 (1.4%)	1 (3.6%)	
	Total Thyroidectomy	56 (62.9%)	53 (76.8%)	20 (71.4%)	
Monolateral Central Compartment lymphadenectomy	No	89 (100%)	68 (98.6%)	28 (100%)	0.426
	Yes	-	1 (1.4%)	-	
Bilateral Central Compartment lymphadenectomy	No	87 (97.8%)	67 (97.1%)	28 (100%)	0.669
	Yes	2 (2.2%)	2 (2.9%)	-	
Monolateral Lateral Compartment lymphadenectomy	No	89 (100%)	69 (100%)	28 (100%)	-
	Yes	-	-	-	
Bilateral Lateral Compartment lymphadenectomy	No	89 (100%)	69 (100%)	28 (100%)	-
	Yes	-	-	-	
Chronic Thyroiditis	No	62 (69.7%)	51 (73.9%)	21 (75%)	0.780
	Yes	27 (30.3%)	18 (26.1%)	7 (25%)	
Variant	Minimally invasive FTC	75 (84.3%)	60 (87%)	23 (82.1%)	0.950
	Encapsulated angioinvasive FTC	10 (11.2%)	6 (8.7%)	4 (14.3%)	
	Widely invasive FTC	4 (4.5%)	3 (4.3%)	1 (3.6%)	
Aggressive Variant	No	85 (95.5%)	63 (91.3%)	25 (89.3%)	0.415
	Yes	4 (4.5%)	6 (8.7%)	3 (10.7%)	
Aggressive Variant on Microfoci	No	24 (96%)	16 (72.7%)	5 (71.4%)	0.068
	Yes	1 (4%)	6 (27.3%)	2 (28.6%)	
AHS on main tumor OR on microfoci	No	85 (95.5%)	58 (84.1%)	23 (82.1%)	0.030
	Yes	4 (4.5%)	11 (15.9%)	5 (17.9%)	
Max Cancer Diameter, mm		22 (16–38)	30 (20–40)	40 (23.5–54)	0.030
N microfoci		1.5 (1–2.5)	1 (1–2)	2 (1–3.5)	0.717
Bilateral	No	73 (84.9%)	57 (89.1%)	21 (84%)	0.710
	Yes	13 (15.1%)	7 (10.9%)	4 (16%)	
Multifocal	No	66 (74.2%)	51 (73.9%)	21 (75%)	0.990
	Yes	23 (25.8%)	18 (26.1%)	7 (25%)	
Lymph Node Metastasis	No	27 (96.4%)	21 (95.5%)	2 (66.7%)	0.101
	Yes	1 (3.6%)	1 (4.5%)	1 (33.3%)	
CC Pathological Lymph Nodes	No	27 (96.4%)	21 (95.5%)	2 (66.7%)	0.101
	Yes	1 (3.6%)	1 (4.5%)	1 (33.3%)	
CC N lymph nodes excised		2 (1–3)	2 (1–4)	3 (2–4)	0.437
CC N Pathological Lymph Nodes		0 (0–0)	0 (0–0)	0 (0–1)	0.147
LC Pathological Lymph Nodes	No	31 (100%)	14 (100%)	7 (100%)	-
	Yes	-	-	-	
LC N lymph nodes excised		0 (0–0)	0 (0–0)	0 (0–0)	0.317
LC N Pathological Lymph Nodes		0 (0–0)	0 (0–0)	0 (0–0)	0.718
Extranodal infiltration	No	76 (100%)	56 (100%)	21 (100%)	
	Yes	-	-	-	
Pathological lymph node max dimension		0 (0–0)	0 (0–0)	0 (0–0)	0.317
Surgical Margin Infiltration	No	89 (100%)	69 (100%)	28 (100%)	-
	Yes	-	-	-	
Extrathyroid Microscopic infiltration	No	87 (97.8%)	68 (98.6%)	28 (100%)	0.706
	Yes	2 (2.2%)	1 (1.4%)	-	
Extrathyroid Macroscopic Infiltration	No	88 (98.9%)	69 (100%)	28 (100%)	0.578
	Yes	1 (1.1%)	-	-	
Vascular-Lymphatic infiltration	No	63 (70.8%)	51 (73.9%)	16 (57.1%)	0.250
	Yes	26 (29.2%)	18 (26.1%)	12 (42.9%)	
pT	1A	7 (7.9%)	8 (11.6%)	2 (7.1%)	0.033
	1B	32 (36%)	11 (15.9%)	3 (10.7%)	
	2	31 (34.8%)	33 (47.8%)	11 (39.3%)	
	3A	18 (20.2%)	17 (24.6%)	12 (42.9%)	
	3B	1 (1.1%)	-	-	
	4A	-	-	-	
pT	pT1 or pT2	70 (78.7%)	52 (75.4%)	16 (57.1%)	0.070
	pT3 or pT4	19 (21.3%)	17 (24.6%)	12 (42.9%)	
pN	0	27 (96.4%)	21 (95.5%)	2 (66.7%)	0.101
	1A	1 (3.6%)	1 (4.5%)	1 (33.3%)	
	1B	-	-	-	
Metastasis	No	86 (100%)	65 (100%)	27 (100%)	-
	Yes	-	-	-	
ATA Risk stratification system	High	7 (7.9%)	5 (7.2%)	2 (7.1%)	0.750
	Intermediate	23 (25.8%)	20 (29%)	11 (39.3%)	
	Low	59 (66.3%)	44 (63.8%)	15 (53.6%)	

BMI, Body Mass Index; N/MNG, nodular/multinodular goiter; CC, Central Compartment; LC, Lateral Compartment; ATA, American Thyroid Association.

**Table 5:** Differences in sociodemographic and pathological characteristics of HCC patients between BMI categories.

		BMI, kg/m^2^			
		<25	25–29.9	>29.9	
		N (%); Median (IQR)	N (%); Median (IQR)	N (%); Median (IQR)	*p* Value
Age at Surgery, years		50 (42–66.5)	56 (51–62)	62 (50.5–73)	0.158
Gender	Female	19 (79.2%)	14 (66.7%)	7 (43.8%)	0.060
	Male	5 (20.8%)	7 (33.3%)	9 (56.3%)	
Hyperthyroidism	No	23 (95.8%)	21 (100%)	16 (100%)	0.457
	Yes	1 (4.2%)	-	-	
Preoperative Diagnosis	Basedow	-	-	-	0.751
	Indeterminate nodule	17 (70.8%)	12 (57.1%)	12 (75%)	
	Malignancy	4 (16.7%)	6 (28.6%)	2 (12.5%)	
	N/MNG	3 (12.5%)	3 (14.3%)	2 (12.5%)	
	Plummer	-	-	-	
Substernal Goiter	No	24 (100%)	20 (95.2%)	15 (93.8%)	0.495
	Yes	-	1 (4.8%)	1 (6.3%)	
Type of Surgery	Completion Thyroidectomy	-	1 (4.8%)	-	0.897
	Lobectomy	5 (20.8%)	3 (14.3%)	3 (18.8%)	
	Lobectomy + Completion Thyroidectomy	1 (4.2%)	1 (4.8%)	1 (6.3%)	
	Total Thyroidectomy	18 (75%)	16 (76.2%)	12 (75%)	
Monolateral Central Compartment lymphadenectomy	No	24 (100%)	20 (95.2%)	16 (100%)	0.380
	Yes	-	1 (4.8%)	-	
Bilateral Central Compartment lymphadenectomy	No	23 (95.8%)	19 (90.5%)	15 (93.8%)	0.768
	Yes	1 (4.2%)	2 (9.5%)	1 (6.3%)	
Monolateral Lateral Compartment lymphadenectomy	No	24 (100%)	20 (95.2%)	16 (100%)	0.380
	Yes	-	1 (4.8%)	-	
Bilateral Lateral Compartment lymphadenectomy	No	23 (95.8%)	21 (100%)	16 (100%)	0.457
	Yes	1 (4.2%)	-	-	
Chronic Thyroiditis	No	16 (66.7%)	17 (81%)	14 (87.5%)	0.268
	Yes	8 (33.3%)	4 (19%)	2 (12.5%)	
Variant	Encapsulated angioinvasive HCC	6 (25%)	3 (14.3%)	5 (31.3%)	0.208
	Minimally invasive HCC	15 (62.5%)	10 (47.6%)	9 (56.3%)	
	Widely invasive HCC	3 (12.5%)	8 (38.1%)	2 (12.5%)	
Aggressive Variant	No	20 (83.3%)	12 (57.1%)	12 (75%)	0.140
	Yes	4 (16.7%)	9 (42.9%)	4 (25%)	
Aggressive Variant on Microfoci	No	6 (100%)	6 (100%)	2 (100%)	-
	Yes	-	-	-	
Max Cancer Diameter, mm		30 (19.5–45)	35 (20–45)	34 (21.5–44.5)	0.910
N microfoci		1 (1–2)	1.5 (1–2.5)	0 (0–0)	0.717
Bilateral	No	19 (79.2%)	17 (81%)	15 (100%)	0.169
	Yes	5 (20.8%)	4 (19%)	-	
Multifocal	No	18 (75%)	15 (71.4%)	15 (93.8%)	0.221
	Yes	6 (25%)	6 (28.6%)	1 (6.3%)	
Lymph Node Metastasis	No	4 (80%)	5 (62.5%)	4 (100%)	0.344
	Yes	1 (20%)	3 (37.5%)	-	
CC Pathological Lymph Nodes	No	5 (100%)	5 (62.5%)	4 (100%)	0.129
	Yes	-	3 (37.5%)	-	
CC N lymph nodes excised		2 (1–3)	3 (2–7)	2.5 (1–5)	0.437
CC N Pathological Lymph Nodes		0 (0–0)	0 (0–1)	0 (0–0)	0.147
LC Pathological Lymph Nodes	No	5 (83.3%)	4 (80%)	3 (100%)	0.719
	Yes	1 (16.7%)	1 (20%)	-	
LC N lymph nodes excised		21 (21–21)	12 (12–12)	0 (0–0)	0.317
LC N Pathological Lymph Nodes		0 (0–0)	0 (0–0)	0 (0–0)	0.718
Pathological lymph node max dimension		0 (0–0)	4 (3–21)	0 (0–0)	0.317
Extranodal infiltration	No	22 (100%)	18 (100%)	13 (100%)	-
	Yes	-	-	-	
Surgical Margin Infiltration	No	24 (100%)	21 (100%)	16 (100%)	-
	Yes	-	-	-	
Extrathyroid Microscopic infiltration	No	22 (91.7%)	17 (81%)	16 (100%)	0.148
	Yes	2 (8.3%)	4 (19%)	-	
Extrathyroid Macroscopic Infiltration	No	23 (95.8%)	20 (95.2%)	15 (93.8%)	0.956
	Yes	1 (4.2%)	1 (4.8%)	1 (6.3%)	
Vascular-Lymphatic infiltration	No	11 (45.8%)	10 (47.6%)	9 (56.3%)	0.790
	Yes	13 (54.2%)	11 (52.4%)	7 (43.8%)	
pT	1A	1 (4.2%)	3 (14.3%)	-	0.752
	1B	6 (25%)	4 (19%)	4 (25%)	
	2	8 (33.3%)	5 (23.8%)	7 (43.8%)	
	3A	8 (33.3%)	8 (38.1%)	4 (25%)	
	3B	1 (4.2%)	1 (4.8%)	1 (6.3%)	
	4A	-	-	-	
pT	pT1 or pT2	15 (62.5%)	12 (57.1%)	11 (68.8%)	0.770
	pT3 or pT4	9 (37.5%)	9 (42.9%)	5 (31.3%)	
pN	0	4 (80%)	5 (62.5%)	4 (100%)	0.476
	1A	-	2 (25%)	-	
	1B	1 (20%)	1 (12.5%)	-	
Metastasis	No	24 (100%)	19 (100%)	16 (100%)	-
	Yes	-	-	-	
ATA Risk stratification system	High	3 (12.5%)	8 (38.1%)	2 (12.5%)	0.220
	Intermediate	11 (45.8%)	6 (28.6%)	6 (37.5%)	
	Low	10 (41.7%)	7 (33.3%)	8 (50%)	

BMI, Body Mass Index; N/MNG, nodular/multinodular goiter; CC, Central Compartment; LC, Lateral Compartment; ATA, American Thyroid Association.

**Table 6:** Univariate and multivariate logistic regression to identify predictors of AHS.

	Univariate			Multivariate		
	OR	95% C.I.	*p*	OR	95% C.I.	*p*
BMI	1.016	1.00–1.03	0.05	1.018	1.01–1.03	0.028
Age at Surgery	1.001	0.99–1.00	n.s.	1.001	0.995–1.01	n.s.
Female Gender	0.816	0.67–0.98	0.036	0.795	0.66–0.96	0.019

BMI, Body Mass Index; OR, Odds Ratio; C.I., Confidence Interval; n.s. not significant.

## Data Availability

Data are contained within the article.
